# How important is the number of pelvic lymph node retrieved to locorregional staging of cervix cancer?

**DOI:** 10.1590/S1679-45082013000400008

**Published:** 2013

**Authors:** Thales Paulo Batista, Artur Lício Rocha Bezerra, Mário Rino Martins, Vandré Cabral Gomes Carneiro

**Affiliations:** 1 Faculdade Pernambucana de Saúde, Instituto de Medicina Integral Professor Fernando Figueira, Recife, PE, Brazil; 2 Hospital de Câncer de Pernambuco, Recife, PE, Brazil

**Keywords:** Uterine cervical neoplasms/surgery, Lymphatic metastasis, Lymph node excision

## Abstract

**Objective:**

To explore how important is the number of pelvic lymph nodes dissected for the nodal staging in FIGO IA2-IB2 cervical cancer, submitted to radical surgical treatment.

**Methods:**

A cross-sectional study was carried out on patients who underwent Piver class II radical hysterectomy and pelvic lymphadenectomy, in two centers in the state of Pernambuco, from January, 2001 to December, 2008. The analysis of the area under the ROC curve was adopted as a summary-measure of discriminatory power of the number of nodes dissected in predicting the pelvic nodal status. Additionally, we also confirm our findings using logistic regression and the Fisher's exact test.

**Results:**

The postoperative pathological study included 662 pelvic lymph nodes dissected (median per-patient=9, q_25_=6 − q_75_=13) from 69 patients. The ROC curve analysis revealed AUC=0.642, for the discriminatory value of the number of nodes dissected in predicting the pelvic nodal status. Similar findings were found after categorization using 10 and 15 lymph nodes as cut-offs (AUC=0.605 and 0.526, respectively). Logistic regression revealed odds ratio of 0.912 (95% CI=0.805-1.032; p=0.125) for the predictive value of the number of nodes dissected, and a number of nodes ≥10 or ≥15 lymph nodes was not significantly associated with the nodal status by the Fischer's exact test (p=0.224 and p=0.699, respectively).

**Conclusion:**

The number of pelvic lymph nodes dissected did not correlate with pelvic lymph node metastatic involvement. This study suggests that dissection of a greater number of lymph nodes does not increase locoregional nodal staging in cervical cancer.

## RESUMO

**Objetivo:**

Avaliar a importância do número de linfonodos pélvicos dissecados para o estadiamento locorregional de pacientes portadoras de câncer do colo uterino com estadiamento FIGO IA2 a IB2, submetidas a tratamento cirúrgico radical.

**Métodos:**

Estudo de corte transversal incluindo pacientes submetidas à histerectomia radical tipo II de Piver e linfadenectomia pélvica, em dois centros pernambucanos, entre janeiro de 2001 e dezembro de 2008. Utilizou-se análise da área sob curva ROC como medida-resumo do desempenho do número de linfonodos dissecados para a predição do acometimento metastático linfonodal pélvico. Adicionalmente, também se avaliou a relação entre essas variáveis, usando a regressão logística e o teste exato de Fisher.

**Resultados:**

A avaliação anatomopatológica incluiu 662 linfonodos dissecados (mediana=9, q_25_=6 – q_75_=13) de 69 pacientes. A avaliação da área sob curvas ROC revelou AUC=0,642 para a predição do estadiamento linfonodal pélvico pelo número de linfonodos dissecados. AUCs de 0,605 e 0,526 foram observadas quando se classificaram as pacientes, utilizando-se 10 e 15 linfonodos como pontos de corte, respectivamente. Por regressão logística, evidenciou-se *odds-ratio* de 0,912 (IC95%=0,805-1,032; p=0,125). A dissecção de ≥10 ou ≥15 linfonodos não se associou ao achado anatomopatológico de comprometimento metastático dos linfonodos pelo teste de Fisher (p=0,224 e p=0,699, respectivamente).

**Conclusão:**

O número de linfonodos dissecados não se correlacionou com comprometimento metastático linfonodal pélvico nessa casuística, o que sugere que a dissecção de um maior número de linfonodos não incremente o estadiamento locorregional do câncer de colo uterino.

## INTRODUCTION

Lymph node metastasis is one of the most important prognostic factors of cervical cancer^(1)^. Thus, in order to elucidate the nodal status and the pattern of lymphatic spread of tumor^(2)^, systematic pelvic lymph node dissection is incorporated as an integral part of surgical procedures recommended to treat early-stage cervical carcinomas^(3)^. However, whether patients suffering of cervical cancer would benefit from systematic lymph node dissection remains unclear.

The radical lymphadenectomy, represented by the number of nodes removed, is supposed to have some therapeutic benefit for patients with operable cervical cancer; whereas a greater number of pelvic lymph node dissected (NPLD) appears to be the best way to improve nodal staging in these patients. Nevertheless, in order to reduce treatment-related morbidity, more tailored and conservative surgical approaches have been proposed to assess lymph node status. Since these methods include the exam of a fewer number of lymph nodes, we reviewed the clinical value of NPLD for the nodal staging in cervix cancer using our data from the Northeastern region in Brazil.

## OBJECTIVE

To explore how important is the number of pelvic lymph node dissected for the nodal staging in patients with IA2 to IB2 cervical cancer.

## METHODS

A cross sectional study was carried out on women newly diagnosed with stage I cervix cancer who underwent radical hysterectomy and pelvic lymph node dissection at the *Hospital de Câncer de Pernambuco* (HCP) and *Instituto de Medicina Integral Professor Fernando Figueira* (IMIP), from January, 2001 to December, 2008. We limited our analysis to adults (≥18 years) with complete data in their medical records. Patients staged IA1 were excluded because of their low rates of lymph node metastasis^(4)^, as well as patients who underwent neoadjuvant radiation therapy or chemoradiation, for which we expected a lower number of lymph nodes examined. Previous history of cancer was also considered a criterion of exclusion for this study. This study protocol was reviewed by our Research Ethics Committee (CAAE: 0045.0.447.000-10).

Baseline characteristics, including the nodal status (metastatic nodes or clear nodes) and the NPLD, were retrospectively assessed according to the postoperative histopathological exam of the hysterectomy and pelvic lymphadenectomy. The prognostic value of NPLD in predicting lymph node status (metastatic nodes or clear nodes) was assessed using logistic regression and receiver operating characteristic (ROC) curve analysis. Logistic regression was applied as odds ratios (OR) and 95%CI. The c-statistic, equivalent to the area under the ROC curve (AUC), was adopted to establish the overall discriminatory power of NPLD in predicting the nodal status. The NPLD was explored as a continuous and categorical variable. In this last analysis, the NPLD was categorized according to clinically relevant cut-offs, as described for an appropriated pathological TNM staging (10 nodes)^(5)^, or as significantly associated with increased survival outcomes according to Rossi et al.^(6)^ (15 nodes). Furthermore, we also applied the chi-square tests (i.e., Yates's correction or Fischer's exact test, as appropriate) to check the association between the categorized NPLD and the nodal status. Statistical analyses were performed using the MedCalc 12.4 statistical package (MedCalc Software, Mariakerke, Belgium), and all analyses considered a two-tailed p-value of 0.05 as statistically significant.

The same surgical team performed all procedures using standard class II radical hysterectomy^(7)^ and pelvic lymph node dissection without para-aortic lymphadenectomy. Radiation therapy was usually offered as adjuvant therapy. This approach included external pelvic radiation therapy (total dose ranging from 45 to 50Gy; 180cGy/day) and vaginal high dose rate (HDR) brachytherapy (total dose of 15Gy). Adjuvant chemoradiation included concurred platinum-based chemotherapy. Platinum- or taxane-based chemotherapy was applied for palliative intent as appropriate.

## RESULTS

Ninety-nine patients suffering of early-stage cervical cancer were initially selected after surgical treatment at our centers (HCP=79% and IMIP=21%) with malignant diagnosis histologically confirmed by preoperative biopsy or conization of the uterine cervix, and only squamous cell carcinoma and adenocarcinoma were found as histological types in this sample. Eleven patients were excluded of our analysis due to incomplete data. We also exclude ten patients staged IA1 as well as nine patients who underwent neoadjuvant radiation (n=7) or chemoradiation therapy (n=2). The median age and pregnancies of eligible patients were 44-year (q_25_=36 – q_75_=52) and 4 (q_25_=2 – q_75_=6), respectively. Among these remaining patients, 26 (37.7%) underwent surgery alone and 43 received some adjuvant therapy (62.3%). Adjuvant radiotherapy was offered because compromised vaginal margin (n=3), lymph node metastasis (n=13), poorly differentiated cervix cancer/ synchronous endometrial adenocarcinoma (n=1) or tumor size >4cm (n=26). Three patients (4.3%) received concurred adjuvant chemotherapy (chemoradiation) because of unsuspected endometrial adenocarcinoma diagnosed on postoperative pathological exam (n=1) or multiple pelvic lymph node metastasis (n=2).

The summary of baseline characteristics according to the postoperative pathological exams from the 69 patients selected to analysis is presented on table 1. These studies included 662 pelvic lymph nodes retrieved from the external iliac, obturator, interiliac, parametrial, and common iliac areas (median=9; q_25_=6 – q_75_=13). (18.8%). Fifty-six (81.2%) patients had no metastatic nodes and Accordingly, the NLPD was ≥10 nodes in 33 patients (47.8%), and ≥15 nodes in 13 only one metastatic node was found in the majority of patients with lymph node metastasis (76.9%).

**Table 1:** Baseline characteristics according to the postoperative pathological exam

Variables		n (%)
Histological type		
	Squamous cell carcinoma	52 (75.4)
	Adenocarcinoma	17 (24.6)
Histological grade		
	G1	24 (34.8)
	G2/3	45 (65.2)
Tumor size, cm		
	<1	24 (34.8)
	≥1	45 (65.2)
Lymph node metastasis		
	Clear nodes	56 (81.2)
	Metastatic nodes	13 (18.8)

**Figure 1:**
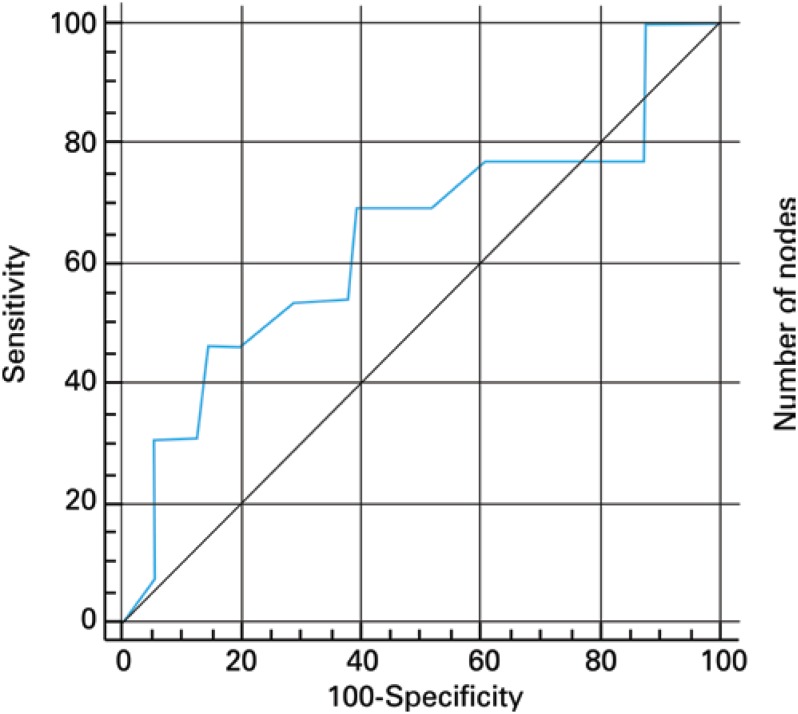
ROC curve for the overall discriminatory power of number of pelvic lymph node dissected in predicting nodal status (AUC=0.642)

**Figure 2:**
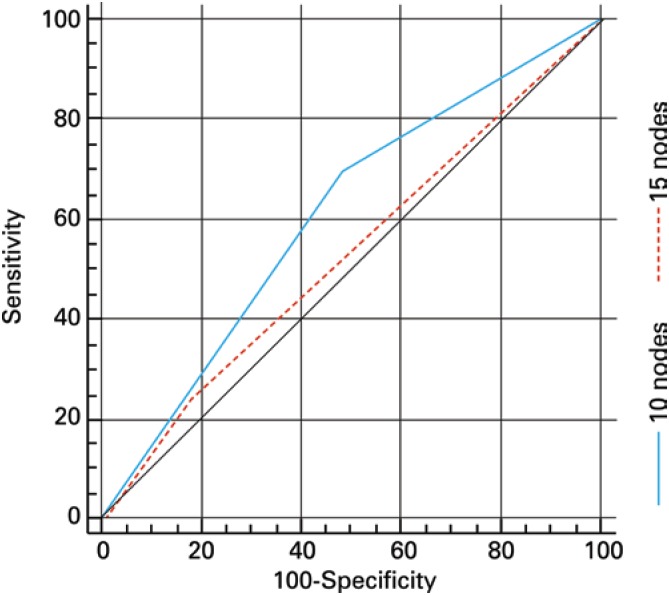
ROC curve for the prognostic value of the number of pelvic lymph node dissected after categorization using 10 and 15 lymph nodes as cut-off points (AUC=0.605 and 0.526, respectively)

Figure 1 shows the ROC curve for the overall discriminatory power of NPLD in predicting nodal status with corresponding AUC of 0.642. AUCs of 0.605 and 0.526 were observed when the NPLD was categorized using 10 and 15 lymph nodes as cut-off points, respectively (Figure 2). Logistic regression revealed OR=0.912 (95%CI=0.805-1.032; p=0.125) for the prognostic value of NPLD in predicting lymph node status, and the NPLD ≥10 or ≥15 lymph nodes was not associated with the pathologic finding of metastatic lymph nodes by Fisher test (p=0.224 and p=0.699, respectively).

## DISCUSSION

Pelvic lymph node dissection is performed by skeletonizing vessels and removing lymph-node containing adipose baring fat tissue. A systematic pelvic lymphadenectomy usually includes the external iliac, obturator, interiliac (internal iliac), parametrial, and common iliac stations. The median number of lymph nodes removed in this standard procedure varies a lot among different authors, ranging from 13 to 48 nodes^(8–12)^, but the median number of lymph nodes examined per patient has also significantly declined over the years^(13)^. Accordingly, the median number of pelvic lymph nodes examined was relatively low in this study.

The incidence of pelvic lymph node metastasis in cervical cancer patients staged IA2-IB2 ranges from 3.7% to 21.7%^(9,14–18)^, depending on many clinical and pathologic factors^(2,19)^. In our data, lymph node metastases were found in 18.8% of cases and only one metastatic node was found in the majority of them (76.9%). Our rate of single lymph node metastasis was higher than previously described by other authors, which reported rates from 11.2 to 49%^(2,16)^.

However, because routine histological examination is usually done on a very limited number of sections, the incidence of histologically defined lymph node metastasis may be lower than the real number^(20)^. Accordingly, Lentz et al.^(21)^ found that micrometastases could be identified in histologically negative lymph nodes in up to 15% of early-stage cancer patients using immunohistochemical methods, which approximates the recurrence rate for patients with negative nodes. Similarly, Juretzka et al.^(22)^ reported immunohistochemically detectable micrometastases in about 8.1% of histologically negative nodes. Thus, because micrometastatic disease represents an independent prognostic factor for patients suffering cervix cancer^(23)^, and patients with greater numbers of lymph nodes analyzed appears more likely to have lymph node micrometastases identified^(21)^, radical lymphadenectomy has been suggested as the best way to improve the nodal staging of patients with cervix cancer.

On the other hand, despite a greater NPLD appears obvious to improve detection rates of lymph node metastasis, we did not find a significant correlation between NPLD and nodal status using logistic regression analysis. Similarly, c-statistic was also applied to establish the overall discriminatory power of NPLD in predicting nodal status. Usually, AUC between 0.8 and 0.9 indicates excellent diagnostic accuracy and an AUC>0.7 should be considered clinically useful. However, according to this approach, the NPLD had a poor diagnostic accuracy in predicting nodal status.

Though our findings may be the result of our lower NPLD, which may have minimized the accuracy of the NPLD in predicting the nodal status, our findings support that replacement of the routine histological examination including the largest possible amount of nodes by a more tailored approach including a few number of nodes (*e. g.*: sentinel lymph node biopsy) does not decrease nodal status detection in cervical cancer. Despite our relative small sample size, the main scientific merit of this study was to explore these important issues using multiple statistical methods, such as logistic regression and c-statistic analysis.

Exploring the incidence and distribution pattern of lymph node metastasis in patients staged IB-IIB cervix cancer, Sakuragi et al.^(2)^ provided some initial basis for determining the site of selective lymph node dissection and observed the obturator lymph nodes were most frequently involved and may be the sentinel lymph nodes of this malignancy. Furthermore, because imaging techniques have limitations in diagnosing microscopic lymph node metastasis in a preoperative setting^(24–26)^, the sentinel lymph node procedure has emerged as an alternative to systematic lymphadenectomy in cervix cancer.

Recent literature supports the safety and feasibility of sentinel lymph node biopsy in gynecologic malignancies and its utility for early-stage cervical cancer remain promising^(27)^. This technique is an accurate method for identifying lymph node metastases in cervix cancer^(27–30)^ and, not surprising, also improves micrometastasis detection^(28)^ and seems to be a more sensitive procedure in detecting pelvic lymph node metastases compared to complete lymphadenectomy^(30)^. Notwithstanding, parametrectomy and side-specific lymphadenectomy remain important components of the surgical management in cases of failed mapping^(28,29)^.

## CONCLUSION

The number of pelvic lymph node dissected did not correlate with the most important prognostic factor for cervix cancer patients – namely, nodal status, in this data from the Northeastern region in Brazil. This study suggests that dissection of a greater number of lymph nodes does not serve to increase the nodal staging in early-staged cervix cancer.
